# Very bright orange fluorescent plants: endoplasmic reticulum targeting of orange fluorescent proteins as visual reporters in transgenic plants

**DOI:** 10.1186/1472-6750-12-17

**Published:** 2012-05-03

**Authors:** David GJ Mann, Laura L Abercrombie, Mary R Rudis, Reggie J Millwood, John R Dunlap, C Neal Stewart

**Affiliations:** 1 Department of Plant Sciences, University of Tennessee, Knoxville, TN, 37996, USA; 2 BioEnergy Science Center, Oak Ridge National Laboratory, Oak Ridge, TN, 37831, USA; 3 Division of Biology, University of Tennessee, Knoxville, TN, 37996, USA

**Keywords:** Endoplasmic reticulum targeting, Fluorescent proteins, GFP, Marker genes, OFP, Orange fluorescent protein, Reporter genes, RFP, Subcellular localization, Transgenic plants, Visual markers

## Abstract

**Background:**

The expression of fluorescent protein (FP) genes as real-time visual markers, both transiently and stably, has revolutionized plant biotechnology. A palette of colors of FPs is now available for use, but the diversity has generally been underutilized in plant biotechnology. Because of the green and far-red autofluorescent properties of many plant tissues and the FPs themselves, red and orange FPs (RFPs, and OFPs, respectfully) appear to be the colors with maximum utility in plant biotechnology. Within the color palette OFPs have emerged as the brightest FP markers in the visible spectra. This study compares several native, near-native and modified OFPs for their “brightness” and fluorescence, therefore, their usability as marker genes in transgenic plant tissues.

**Results:**

The OFPs DsRed2, tdTomato, mOrange and pporRFP were all expressed under the control of the CaMV *35S* promoter in agroinfiltration-mediated transient assays in *Nicotiana benthamiana*. Each of these, as well as endoplasmic reticulum (ER)-targeted versions, were stably expressed in transgenic *Nicotiana tabacum* and *Arabidopsis thaliana*. Congruent results were observed between transient and stable assays. Our results demonstrated that there are several adequate OFP genes available for plant transformation, including the new pporRFP, an unaltered tetramer from the hard coral *Porites porites*. When the tandem dimer tdTomato and the monomeric mOrange were targeted to the ER, dramatic, ca. 3-fold, increase in plant fluorescence was observed.

**Conclusions:**

From our empirical data, and a search of the literature, it appears that tdTomato-ER and mOrange-ER are the two highest fluorescing FPs available as reporters for transgenic plants. The pporRFP is a brightly fluorescing tetramer, but all tetramer FPs are far less bright than the ER-targeted monomers we report here.

## Background

Since the discovery and isolation of the green fluorescent protein (GFP) from the Pacific jellyfish *Aequorea victoria*, fluorescent proteins (FPs) have become an increasingly powerful tool for use in molecular biology [1-3]. The lack of a required substrate or co-factor along with the visible fluorescence that is emitted upon excitation of the fluorophore make FPs desirable tools and reporters for a wide variety of biological applications. Recent advances in imaging methods have also enhanced the applications of FPs in plant biology [4]. In plant gene expression studies, the genes for FPs are often overexpressed alone or fused directly to other genes of interest to monitor spatial expression patterns, and entire vector sets have been constructed for the ease of this application [5,6]. Additionally, FPs have been used as tracking agents to detect and improve the efficiency of transient expression and stable plant transformation systems. In this case, FPs are typically under the transcriptional regulation of highly constitutive promoters such as maize ubiquitin 1 promoter (*ZmUbi1*) or cauliflower mosaic virus (CaMV) *35S* promoter [7,8]. While whole cell or whole organism expression of FPs are common, fluorescent reporter genes have also been cloned under the control of tissue-specific promoters for discrete expression in transgenic plants, including in pollen [9,10], endosperm and aleurone cells [11-13], roots [14,15] and vascular tissues [16,17]. FPs are useful to characterize inducible promoters [18] and can also be fused directly to sequence peptide tags at the N- or C-terminus of a gene sequence and targeted intracellularly to specific studies [19].

Although other FPs have been added to the color palette during the past 12 years, GFP has remained the most commonly used FP in these studies; GFPs generally form monomers in physiologically-relevant concentrations overexpressed in the cytosol, whereas native coral-derived FPs (yellow through far red) tend to autotetramerize. Nevertheless coral-derived FPs have gained wider use in recent years in transgenic plant studies simply because many of them are brighter, owing partially to their longer wavelengths. The longer wavelengths needed to excite RFPs also have much lower levels of autofluorescence in mature green tissue as compared to UV or blue light, which is used to visualize GFP [1]. The greatest source of autofluorescence interference is chlorophyll autofluorescence (flue light excitation) in green tissues, which can obscure GFP fluorescence.

DsRed, derived from coral *Discosoma* sp. was the first coral-derived FP to be used in plants as a reporter gene [20,21], and remains the most widely-used FP in biology after GFP. Systematic mutations have since been introduced into DsRed to improve its folding dynamics, solubilization, photostability and to render monomerization, and more recent mutations and improvements in DsRed have yielded derivative FPs with increased fluorescence intensity or brightness (e.g. increased extinction coefficient, quantum yield) and altered spectral properties (e.g. shifted excitation and emission wavelengths) for reporter gene applications [2]. These include mRFP1 [22] along with tdTomato, mStrawberry, and, mOrange [2,23]. Furthermore, additional sources of coral- and other organism-derived FPs beyond *Discosoma* sp. are constantly being discovered and have recently been exploited to produce novel FPs, potentially resulting in improved FP reporter genes for plant biotechnology [24]. Another gene in the toolbox is mEosFP, which has recently been used in plants in various organelle-targeted versions [25]. EosFP is a green-to-red (actually orange) photoconvertable FP that fluoresces green when by blue light. When excited by 390 nm-405 nm light for a few seconds will convert to orange emission (581 nm maximum).

The aim of this study was to survey a sample of promising FPs that have seldom been used in plants to compare their performance as reporter genes. We also set out to improve them for use in applications where whole-plant fluorescence is of paramount importance (e.g., detecting inducible expression). We evaluated and modified the following FPs (maximal excitation and emission wavelengths in nm): DsRed2 (563, 582), tdTomato (554, 581) mOrange (548, 562) and pporRFP (578, 595). Whereas many of these proteins are often called red fluorescent proteins (RFPs), as Shaner et al. [3] rightly point out, their emissions are all orange. Therefore we will refer to these proteins as orange fluorescent proteins (OFPs). The extinction coefficients and quantum yields of these OFPs indicated that each should be useful as markers in plants, but all started with less brightness than tdTomato: DsRed2 (38% as bright), mOrange (52% as bright), and pporRFP (56% as bright). Since we are especially interested in their use as transgenic markers, and not as fusion protein candidates, tetramerization was not deemed to be a negative factor. However, we did use some monomeric protein-coding variants chosen because of their brightness. We compared non-targeted and endoplasmic reticulum- (ER-) targeted variants under the control of a constitutive promoter in identical DNA vector backbones. Transient expression in *Nicotiana benthamiana* using agroinfiltration and stable transgenic *Arabidopsis thaliana* and tobacco (*Nicotiana tabacum*) plants were assayed using epifluorescence and confocal microscopy, and spectrofluorescence measurements.

## Results and discussion

### Agroinfiltration-mediated transient expression of OFPs

**Figure 1:**
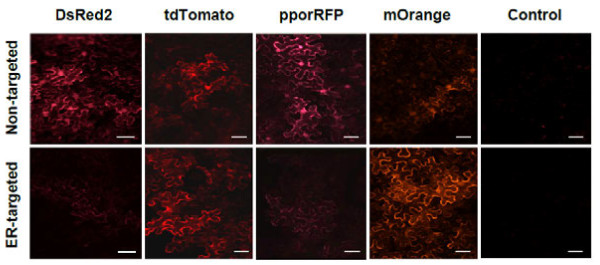
**Representative confocal microscopy images showing a comparison of fluorescence in tobacco leaves following agroinfiltration with *A. tumefaciens*strain GV3850 containing constructs expressing DsRed2, DsRed2-ER, tdTomato, tdTomato-ER, pporRFP, pporRFPmut-ER, mOrange, mOrange-ER and empty vector controls.** Images were taken using scanning confocal microscopy at 48 hours after agroinfiltration. Scale bar represents 50 μm.

The agroinfiltration experiment in *Nicotiana benthamiana* was designed to rapidly screen the expression vectors for functionality, but to also assay gross comparisons of the effect of ER-targeting, e.g., adding a signal peptide fusion to the N-terminus and an HDEL ER retention signal to the C-terminus that successfully improved whole plant fluorescence for GFP [26]. We were initially perplexed that the apparent fluorescence varied among OFPs, instead of increasing the fluorescence in all the four different OFP genes. Notable fluorescence increase from ER-targeting was observed only for the non-tetramers: tdTomato (tandem-dimer Tomato), which essentially forms a monomer OFP, and the monomeric variant, mOrange (Figure 1). The tdTomato is a head-to-tail dTomato variant with a 16 amino acid linker, and therefore contains two chromophores, which explains why it is approximately twice as bright as the other OFPs [3,18]. With a large Stokes shift of 27 nm, tdTomato is also practically easier to visualize and measure compared with the other OFPs tested with lower Stokes shifts (between 14 nm and 19 nm). We hypothesize that the additions to the N- and, especially the C- termini altered the homotetramerization of DsRed-2 and pporRFP, which could have affected both their accumulation and ultimately their fluorescence. The agroinfiltration data also appeared to be congruent with the spectrofluorometric data of the stable transformants described below.

### Stable expression of unaltered OFP genes in tobacco

**Figure 2:**
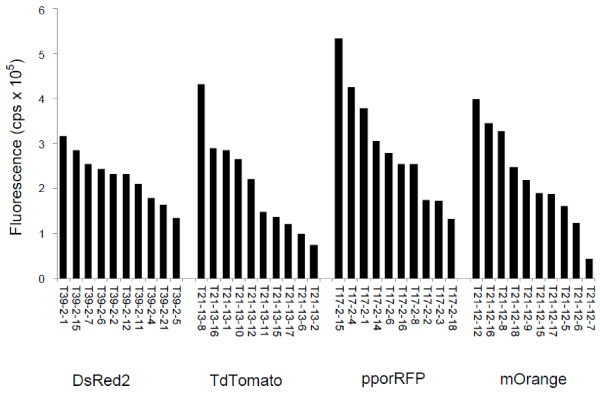
**Comparison of different OFP expression in tobacco.** Ten independent transgenic lines overexpressing each FP gene construct were selected. Leaf samples (youngest fully expanded leaf) from each of three individual plants were measured for each transgenic line and the average fluorescence intensity at the peak excitation wavelength is shown. All fluorescent measurements were normalized to the non-transgenic tobacco control (cv. Xanthi).

Whereas agroinfiltration is useful as a first-pass gross screen for cassette functionality, stable transformation more closely mimics the range of expression levels seen when gene constructs are integrated in variable insertion sites. Stable expression was required in order to quantitatively compare the expression levels of the different fluorescent protein encoding genes in similar tissues. Transformants (up to 10 events) of the most fluorescent T_1_ transgenic tobacco (*Nicotiana tabacum* cv. Xanthi) lines were selected for analysis and, therefore, microscopy and fluorospectroscopy analysis of these lines yield a realistic variation with regards to the range of fluorescence in these eudicot species. There was a wide range of fluorescent intensity for the different transgenic lines for each OFP construct, with up to an eight-fold difference from the highest to the lowest expressing line (Figure 2). However, the overall range and average of fluorescence levels for each construct of DsRed2, tdTomato, pporRFP and mOrange were not significantly different, even though there were differences among specific lines within constructs (genes). Therefore, the results demonstrate that selecting any of these FP genes for use as transgenic reporters is justified and each should give relatively congruent results. We did note that the maximum fluorescence among all FP genes and events analyzed was for a pporRFP line (T17-2-15; statistically significant at the P = 0.0001 level) (Figure 2). Also, pporRFP is a unique OFP to transgenic plants; these are the first published quantitative data on its use in plants. It is a tetramer OFP that was originally cloned from the hard coral *Porites porites*; it was chosen for assessment because of its attractive spectral properties [24], which enables microscopy using the standard OFP-DsRed filter sets in epifluorescence microscopy. It was because of these early initial results that we chose pporRFP to include as the scorable FP marker when we constructed the versatile large pANIC vector set for Gateway-enabled monocot transformation [27]. While there are no published expression- or fluorescence data yet in monocots, we observed that pporRFP is very effective in rice (*Oryza sativa*) and switchgrass (*Panicum virgatum*) as a transgenic reporter gene [27,28]. Indeed, until recently, switchgrass transformation has been very difficult and inefficient. The use of overexpression of pporRFP and tracking analysis of transformed tissue has enabled important increases in transformation efficiency of switchgrass [29]. We chose pporRFP instead of tdTomato for this purpose since the latter gene is approximately twice the size of the former and we valued minimizing the total vector size for this project.

### Plant codon optimization effects of pporRFP in stable transgenic plants

**Figure 3:**
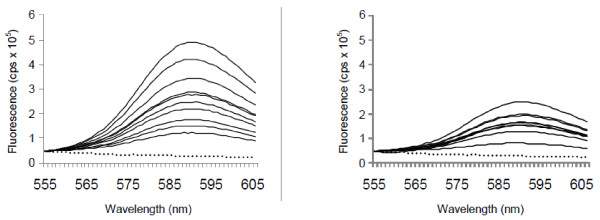
**Fluorescence measurements for tobacco overexpressing pporRFP (A) and pporRFPmut (codon altered) (B).** Ten individual transgenic lines were selected for overexpression of each of the constructs (pporRFP and pporRFPmut). Leaf samples (youngest fully expanded leaf) from each of three individual plants were measured for each transgenic line and the average fluorescence intensity is shown. The non-transgenic tobacco (Xanthi, negative control) is denoted with a dotted line. All fluorescent measurements were normalized to the non-transgenic (Xanthi) control.

As a result of this high fluorescence using the native pporRFP gene, we modified the nucleotide sequence with the intention of optimizing the codons for plants to further enhance the fluorescent expression levels. Utilizing *Arabidopsis thaliana* most commonly used codons as a model, pporRFPmut was stably transformed into tobacco and transgenic T_0_ lines were screened for fluorescence. Ten transgenic lines were selected and carried through to the T_1_ progeny as above, where they were directly compared to the native pporRFP gene for expression of fluorescence. Surprisingly, the overall fluorescence level of the codon-altered pporRFPmut was significantly lower than that of the native pporRFP (Figure 3). The average fluorescent measurements at the peak emission wavelength (595 nm) for the pporRFP and pporRFPmut transgenic tobacco were 2.9 × 10^5^ (± 1.2 × 10^5^) and 1.7 × 10^5^ (± 0.5 × 10^5^), respectively. We are curious as to why plant codon optimization failed, since it has increased expression and accumulation of other proteins synthesized in plants, most notably *Bacillus thuringiensis* endotoxins [30]. One speculation is that in fact perhaps, the codon-optimized genes were overexpressed, yet misfolded, which was observed when DsRed and the RFP eqFP611 were overexpressed in bacteria [31]. Lower expression, in this situation, led to improved fluorescence.

### ER-targeting greatly enhances the fluorescence of tdTomato and mOrange in stable transgenic plants

**Figure 4:**
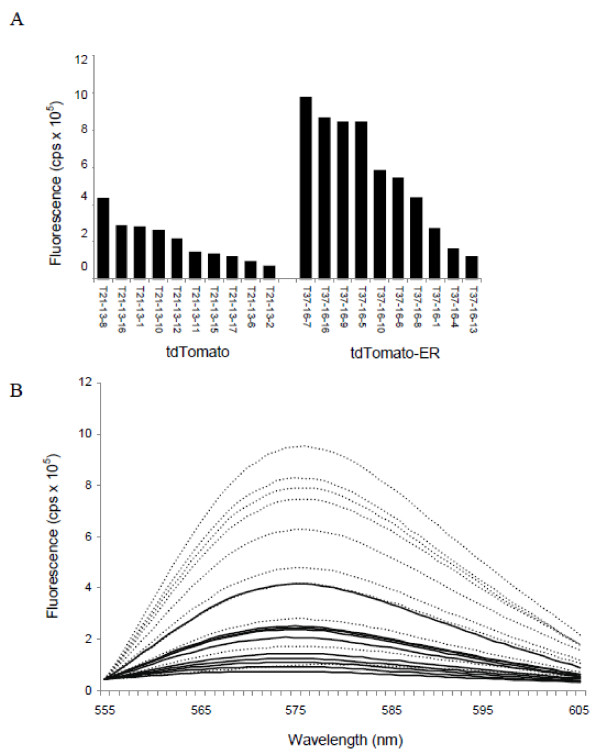
**Fluorescence measurements for tobacco overexpressing tdTomato and tdTomato-ER.(A)** Fluorescence measured at the peak excitation wavelength (581 nm) for each transgenic line. **(B)** Broad fluorescent intensity measured between 555 nm and 605 nm for tdTomato (solid lines) and tdTomato-ER (dotted lines). Ten independent transgenic lines were selected for overexpression of each of the constructs (tdTomato and tdTomato-ER).Leaf samples (youngest fully expanded leaf) from each of three individual plants were measured for each transgenic line and the average fluorescence intensity is shown. All fluorescent measurements were normalized to the non-transgenic (Xanthi) control.

The most important finding of our study is the dramatic enhancement of fluorescence in overexpressed FP genes from the additions of signal peptide and the ER retention peptides to both tdTomato and mOrange. The spectrofluorometric measurements we observe in stably transgenic tobacco expressing tdTomato-ER versus non-targeted tdTomato are congruent with the microscopy images we observed for the agroinfiltration experiments, in which ER retention increased fluorescence (Figures 1 and 4). ER targeting rendered a doubling of fluorescence for tdTomato when examining the best performing lines of each (Figure 4). When we compared the top four lines of each construct relative to fluorescence, there was, on average, nearly a tripling of fluorescence for ER-targeted vs. non-targeted tdTomato (8.9 × 10^5^ (± 0.6 × 10^5^) and 3.2 × 10^5^ (± 0.8 × 10^5^, respectively)). Furthermore, the distributions of fluorescence between the two gene variants have very little overlap (Figure 4), inferring that the use of tdTomato-ER for a reporter gene in transformation studies will enable researchers to identify expression over a greater range in plants, possibly enabling greater visualization and tracking of expressing transgenics. For phytosensing applications or other assays where real-time visible assays of inducible promoters are important, this dramatic increase in fluorescence will be a key feature in success in both the lab and field settings [18,32,33].

**Figure 5:**
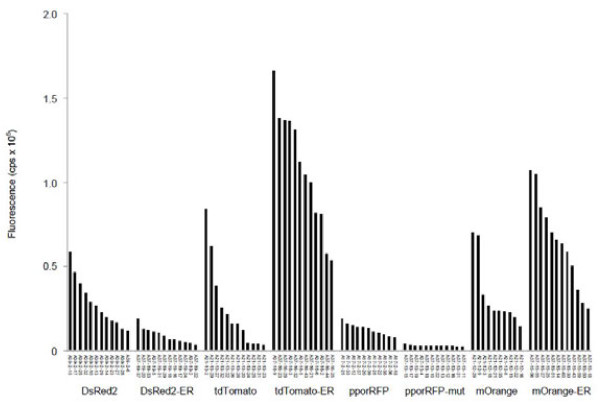
**Comprehensive comparison of OFP fluorescence using spectrofluorometry in *Arabidopsis thaliana *.** Ten to twelve independent transgenic lines of *Arabidopsis* overexpressing each OFP gene construct were selected. Separate measurements on intact rosettes from each of three individual plants were taken for each transgenic line and the average fluorescence intensity at the peak excitation wavelength is shown. All fluorescent measurements were normalized to the wild type control ecotype (Columbia).

**Figure 6:**
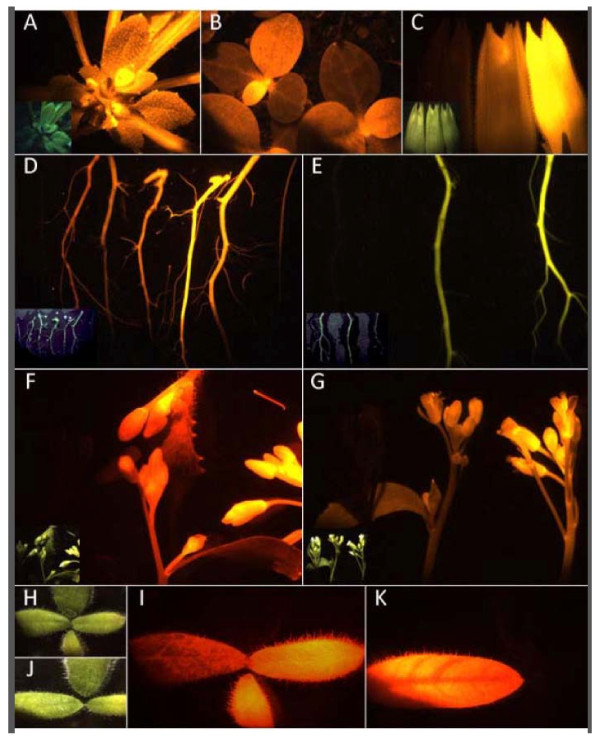
**Representative microscopy images showing a comparison of fluorescence of T**_**2**_*** Arabidopsis *(A,D-G) and T1 tobacco (B, C, H-K) tissue expressing DsRed2, DsRed2-ER, tdTomato, tdTomato-ER, pporRFP, pporRFPmut-ER, mOrange, mOrange-ER or non-transgenic controls.** Transgenic line designations precede species and OFP identities. Images were taken using an epifluorescence microscope. Fluorescent pictures were taken with tdTomato filter set (ex: 535/30 nm, em: 600/50 nm) for all constructs except mOrange, which utilized a mOrange filter set (ex: 535/30 nm, em:585/40 nm). Inset pictures and H and J were taken using white light. **(A)** A37-16-23 potted *Arabidopsis* expressing tdTomato-ER (20 s exp), white light inset 5.5 ms exp. **(B)** Various tobacco line seedlings in potting media expressing pporRFP (2 min exp). **(C)** Tobacco buds expressing (left to right) non-transgenic, T21-12-16 mOrange, T37-15-2 mOrange-ER (20 s exp), white light inset 1.6 ms exp. **(D)***Arabidopsis* roots expressing (left to right) non-transgenic, A17-2-25 pporRFP, A37-14-13 pporRFPmut-ER, A21-13-29 tdTomato, A37-16-23 tdTomato-ER, A39-2-17 DsRed, A37-19-33 DsRed-ER (40 s exp), white light inset 16 ms exp. The root furthest to the right (7^th^) is not clearly shown in the inset. **(E)***Arabidopsis* roots expressing (left to right) non-transgenic, A21-12-9 mOrange, A37-15-56 mOrange-ER (40 s exp), white light inset 16 ms exp. **(F)***Arabidopsis* floral buds expressing (clockwise, starting at 12:00) DsRed, tdTomato, pporRFP, non-transgenic (20 s exp), white light inset 4 ms exp. **(G)***Arabidopsis* flowers expressing (left to right) non-transgenic, mOrange, mOrange-ER (20 s exp), white light inset 2.5 ms exp. **(H,I)** Tobacco leaves clockwise from the top: non-transgenic, T17-2-4 pporRFP, T39-2-1 DsRed, T21-13-8 tdTomato (30 s exp), white light inset 3 ms exp. **(J,K)** Tobacco leaves clockwise from the top: non-transgenic, T37-19-1 DsRed-ER and T37-16-9 tdTomato-ER), white light inset 3 ms exp.

We overexpressed the entire collection of genes (except for pporRFP-ER) in *Arabidopsis*, grown at the same time in similar to conditions to directly compare fluorescence among variants in a common species (Figure 5). Again, the quantitative data are consistent with the qualitative data from the agroinfiltration experiment (Figures 1,4, and 5). There is a large and significant ER-targeting enhancement for tdTomato and mOrange, while the alteration of the tetrameric protein encoding genes pporRFP and DsRed resulted in diminished fluorescence and, hence, usability of these modified genes as reporters in transgenic plants (Figure 6). We observed an approximate 1.5-fold greater fluorescence of *Arabidopsis* compared with tobacco (e.g., see tdTomato data in Figures 4 and 5), but these data were taken at different times and might represent environmental effects or simply inherently brighter tissues (meristems (*Arabidopsis*) vs. leaves (tobacco)) , so we caution against making definitive conclusions regarding direct OFP expression in tobacco vs. *Arabidopsis*. We did, however, observe a similar trend of dramatic enhancement for ER-targeting of mOrange that we observed in tdTomato. When stably expressed in *Arabidopsis*, we observed a 2-fold enhancement of fluorescence in ER-targeted mOrange compared with the non-targeted counterpart (6.5 x 10^5^ (± 2.7 × 10^5^) and 3.3 x 10^5^ (± 2.0 × 10^5^, respectively). Additionally, the ER-targeted tdTomato resulted in more than a 4-fold increase in fluorescence as compared to the non-targeted tdTomato (10.8 × 10^5^ (± 3.5 × 10^5^) and 2.4 x 10^5^ (± 2.5 × 10^5^, respectively). For recombinant proteins that have been shown to be relatively stable when synthesized in the cytosol, ER-targeting and retention has also shown a three-fold increase [34]. ER-targeting via N–terminus signal peptide addition and ER retention via C-terminus HDEL or KDEL addition has been a common tool to aid protein accumulation in transgenic cells [35-38]. The reason for increased fluorescence of the ER-targeted OFPs is likely owed to increased accumulation of protein, which, in the ER, comprises a protected environment with attenuated proteolytic activity that also is imbued with molecular chaperones for protein folding.

## Conclusions

OFPs have emerged as the real-time *in vivo* reporter genes of choice for plant transformation. Endoplasmic reticulum targeting allows the accumulation of greater OFP monomers than non-targeting in select OFPs. TdTomato-ER is the most brightly fluorescing FP marker gene ever characterized in transgenic plants, followed by mOrange-ER. These OFP variants will be especially valuable in quantifying inducible expression in plant organs.

## Methods

### Vector construction and *Agrobacterium* transformation

The coding region of pporRFP was amplified from the vector pGem-T-gbr15 using the forward primer 5′-ATGGCTCTTTCAAAGCAAAGTGG-3′ and reverse primer 5′- TTAGTGATGGTGATGGTGATGGG-3′. mOrange and tdTomato containing vectors were obtained from the laboratory of Roger Tsien (University of California San Diego). mOrange CDS was amplified from pRSET-mOrange using the forward primer 5′- ATGGTGAGCAAGGGCGAGGAGAATA-3′ and reverse primer 5′ TTACTTGTACAGCTCGTCCATGC-3′. The tdTomato CDS was amplified from pRSET-tdTomato using the forward primer 5′- ATGGTGAGCAAGGGCGAGGAGGT-3′ and reverse primer 5′-TTACTTGTACAGCTCGTCCATGC -3′. The resulting products were cloned into the entry vector pCR8/GW-TOPO (Invitrogen). DsRed2 coding sequence (Clontech) was recombined into the entry vector pDONR/Zeo from pET160/GW-DsRed2 using BP Clonase (Invitrogen). The N-terminal signal polypeptide sequence (MKTNLFLFLIFSLLLSLSSAEF) and C-terminal ER-retention polypeptide sequence (HDEL) were added to coding sequences through assembly and amplification PCR as described by Richardson et al [39]. Common 5′assembly primer 5′ER01 (5′-CACCATGAAAACTAATCTTTTCTTGTTTCTTATCTTTTCACTTCTTTTGAGCTTAAGCTCTGCAG-3′) and 3′ assembly primer 3′ER20 (5′- TTACAACTCGTCATGCTTGTACAGCTCGTCCATGCCG-3′) were used in conjunction with sequence specific assembly primers in assembly PCR from a template of fluorescent protein coding sequence described above. Sequence specific assembly primers were as follows: mOrangeER (5′- GGCCATGTTATTCTCCTCGCCCTTGCTCACGAACTCTGCAGAGCTTAAGCTCAAAAGAA-3′), tdTomatoER (5′- CTCTTTGATGACCTCCTCGCCCTTGCTCACGAACTCTGCAGAGCTTAAGCTCAAAAG-3′), DsRed2ER (5′- GATGACGTTCTCGGAGGAGGCGAACTCTGCAGAGCTTAAGCTCAAAAGAA). A mutagenized ppor sequence was created using the GeMS program [40], utilizing *Arabiopsis thaliana* codon usage as a template, and a codon cutoff frequency of 0.2. Full length mutatgenized ppor product was assembled from partially overlapping 60-mer oligos designed via the program Gene Design [39]. All products of assembly PCR were cloned into the directional entry vector pENTR/D-TOPO.

OFP-containing entry vectors were recombined into the plant binary destination vector pMDC32 [3]. Features of this vector include constitutive expression of the gene of interest via a dual CaMV 35 S promoter and hygromycin selection of transgenic plant tissue. Binary vectors were transformed into *Agrobacterium tumefaciens* GV3850. See the Additional file 1 for vector construction diagrams. All nine expression vectors are available via MTA (See http://plantsciences.utk.edu/stewart.htm) as follows: mMDC32-DsRed2, mMDC32-tdTomato, mMDC32-mOrange, mMDC32-pporRFP, mMDC32-pporRFP-mut, mMDC32-DsRed2-ER, mMDC32-tdTomato-ER, mMDC32-mOrange-ER, and mMDC32-pporRFP-mut-ER.

### Plant transformation

Agroinfiltration of *Nicotiana benthamiana* was performed as described by Liu et al. [28] Stable transformation of tobacco cv Xanthi was performed using the Horsch et al.[41] method. Stable transformation of *Arabidopsis* Col1 ecotype was performed using the floral dip method [42]. *Arabidopsis* plants were grown in growth chambers and allowed to self-fertilize. Spectrofluorometry analysis was completed on *Arabidopsis* T_2_ generation seeds, screened on MSA media containing 50 mg/L hygromycin, resistant plants were transferred to potting media and grown in growth chambers (10 hr day length, 18°C/14°C day/night). Plants were 9-week-old rosettes when spectrofluometry was performed. Self-fertilized tobacco plants were grown in the greenhouse (16 hr. day, 27-30°C). Tobacco plants for spectrofluorometry analysis were started as T_1_ segregating seeds grown in potting media, screened for fluorescent protein expression using microscopy, transplanted to individual pots and grown to six-weed old stage under greenhouse conditions.

### Epifluorescent and confocal microscopy and spectrofluorometry

Epifluorescent microscopy of plants was performed using the tdTomato filter set: 535/30 nm excitation and 600/50 nm band pass emission or the mOrange filter set: 535/30 excitation and 585/40 nm band pass emission filter (Olympus stereo microscope model SZX12, Olympus America, Center Valley, PA, USA). Confocal microscopy images were produced using a Leica TCS SP2 microscope (Buffalo Grove, IL. USA), which allows for adjustable bandwidths for the detected fluorescence. The samples were excited with a 543 nm HeNe laser and fluorescence emission was collected from 555–604 nm for mOrange, 570–620 nm for DsRed and tdTomato, and 590 – 610 nm for pporRFP. Chlorophyll autofluorescence was checked for each sample by exciting the sample with 488 nm light from an argon ion laser and collecting emission from 650–750 nm. When chlorophyll autofluorescence was imaged along with the fluorescent protein, images were collected using sequential scanning to prevent bleedthrough fluorescence. Fluorescence measurements (i.e., those results displayed in Figures 2,3,4 and 5) were made using spectrofluorometry according to methods described by Millwood et al. [43] but with an updated Fluorolog®-3 system (Jobin Yvon and Glen Spectra, Edison, NJ, USA). For each of the samples, the youngest fully expanded leaf was chosen to control for developmental stage.

### Statistical analysis

Transgenic plants were statistically analyzed using a one-way analysis of variance in SAS where the response variable was fluorescence measurements from spectrofluorometry. If significant differences were found, mean separations were performed using Fisher’s LSD to determine which genotypes were significantly different at the P = 0.05- to P = 0.0001 levels.

## Competing interest

The authors declare that they have no competing interest.

## Authors’ contributions

DGJM designed experiments, performed some of the fluorospectroscopy experiments analyzed data and drafted most of the manuscript. LLA and MRR designed experments and performed most of the research. RJM performed some of the fluorospectroscopy experiments and statistically analyzed the data. JRD performed confocal microscopy imaging. CNS conceived and coordinated the study, drafted a portion of the manuscript and assisted with revisions. All authors read and approved the final version of this manuscript.

## Supplementary Material

Additional file 1 **Schematic diagram of the T-DNA used in tobacco and Arabidopsis transformation.** The vector shown is the pMDC32-tdTomato-ER. Sequence comparison of native and codon-optimized pporRFP. Underlined sequence represent ER targeting (5′) and ER retention signals (3′). Click here for file
