# Supplementary material for: Very bright orange fluorescent plants: endoplasmic reticulum targeting of orange fluorescent proteins as visual reporters in transgenic plants
Source: BMC Biotechnol. 2012 May 3;12:17. doi: 10.1186/1472-6750-12-17 (PMC3443454; doi:10.1186/1472-6750-12-17)
Supplement: Additional file 1 — Schematic diagram of the T-DNA used in tobacco and Arabidopsis transformation. The vector shown is the pMDC32-tdTomato-ER. Sequence comparison of native and codon-optimized pporRFP. Underlined sequence represent ER targeting (5′) and ER retention signals (3′). [file 1472-6750-12-17-S1.rtf]

Very bright orange fluorescent plants: endoplasmic reticulum targeting of orange fluorescent proteins as visual reporters in transgenic plants


David GJ Mann1,2, Laura L Abercrombie1,2, Mary R Rudis1, Reggie J Millwood1, John R Dunlap3, C  Neal Stewart, Jr1,2*


1Department of Plant Sciences, University of Tennessee, Knoxville, TN 37996, USA
2BioEnergy Science Center, Oak Ridge National Laboratory, Oak Ridge, TN 37831 USA
3Division of Biology, University of Tennessee, Knoxville, TN 37996, USA
*Corresponding author, email nealstewart@utk.edu


Additional File


Figure S1.  Schematic diagram of the T-DNA used in tobacco and Arabidopsis transformation.  The vector shown is the pMDC32-tdTomato-ER.


Figure S2.  Sequence comparison of native and codon-optimized pporRFP. Underlined sequence represent ER targeting (5') and ER retention signals (3').
